# Supplementary material for: A remote measurement study of PTSD and cannabis use among veterans: Recruitment, retention, and data availability
Source: PLoS One. 2025 Sep 29;20(9):e0332239. doi: 10.1371/journal.pone.0332239 (PMC12478881; doi:10.1371/journal.pone.0332239)
Supplement: S1 File — (DOCX) [file pone.0332239.s001.docx]

**Response to Reviewers**
Manuscript ID: PONE-D-25-14945
Title: A remote measurement study of PTSD and cannabis use among veterans – recruitment, retention, and data availability

Dear Dr Kobeissy,

We sincerely thank you for your thorough and constructive feedback on our manuscript. We appreciate the opportunity to revise our submission and have addressed each of the editorial and reviewer comments below. All changes have been made in the revised manuscript, which is provided in both tracked and clean formats.

**Editorial Requests**

**Comment**: Manuscript style and file naming

Response We have ensured the manuscript meets PLOS ONE’s formatting requirements and used appropriate file naming conventions.

**Comment**: Financial disclosure revision

Response: The revised financial disclosure now includes: “The funders had no role in study design, data collection and analysis, decision to publish, or preparation of the manuscript.”

**Comment**: Competing interests statement

Response: We confirm: The revised Conflict of Interest statement now includes: “This does not alter our adherence to PLOS ONE policies on sharing data and materials.”

**Comment**: Data availability and access

Response: There are ethical restrictions on sharing a de-identified dataset due to participant privacy and data sensitivity regarding PTSD and cannabis use. These restrictions were imposed by the University of Southern California Institutional Review Board (IRB #UP-20-00853). Data access requests may be sent to the corresponding author.

We have updated the Data Availability Statement on the cover sheet to reflect this and clarified in the manuscript that we are open to sharing aggregated/anonymised data upon request and IRB approval. It is important to stress, that although we are unable to upload raw participant-level data, we have provided relevant summary tables in the revised Supplementary Information. These include aggregated retention, passive data completion, and wearable usage statistics.

**Comment**: Supporting information captions inclusion

Response: Captions for all supporting information file (item A) have been added at the end of the manuscript, and in-text citations have been updated accordingly.

**Comment**: Reference list review

Response: We have reviewed the reference list and corrected minor formatting errors. We confirm that no retracted articles are cited.

**Reviewer Comments**

**Reviewer #1**

**Comment**: This addresses a critical gap in leveraging digital tools to monitor PTSD and cannabis use among veterans. Strengths include a mixed-methods approach (qualitative insights complementing quantitative data) and rigorous reporting of recruitment/retention metrics. Below are suggestions for improvement:

Response: We are thankful to the reviewer for taking the time to review our manuscript and the helpful comments to improve our manuscript.

**Comment:** Ensure supplementary materials include aggregated data (e.g., summary tables) if raw data cannot be shared. Specify whether de-identified datasets will be archived in a repository post-publication.

Response*:* We have added the Data Availability statement and clarified in the manuscript that, while raw data cannot be publicly shared, aggregated and summary-level data are available in the Supporting Information. A contact for data requests has also been provided (Methods, page 7).

**Comment:** Discuss potential selection bias (e.g., tech-savvy participants) and the short follow-up period (3 months). Acknowledge that feasibility in this sample may not generalize to broader veteran populations.

Response*:* We now explicitly address potential selection bias due to digital access and engagement and limitations of the short follow-up window. This has been added to the Limitations section (Discussion, page 18).

**Comment**: Elaborate on how passive/active data integration can inform clinical interventions (e.g., real-time alerts for symptom escalation). Highlight practical steps for implementing these tools in care settings.

Response*:* We have expanded the Discussion to explain how passive data (e.g., heart rate, sleep) and active data (e.g., self-report scales) can be combined to identify symptom escalation or changes in behaviour. We include potential applications such as triggering clinical alerts or enabling real-time monitoring in care settings (Discussion, page 18).

**Comment:**  Address minor grammatical errors and simplify complex sentences (e.g., in the Abstract and Introduction).

Response*:* We have reviewed and corrected minor grammatical issues throughout the manuscript, including the Abstract and Introduction. Several complex sentences were simplified for clarity.

**Reviewer #2**

**Comment:** Quite an interesting topic and a unique demography considering the rate at which PTSD impacts the veterans, albeit age or length of service. PTSD has been linked to several other neurodegenerative and cardiovascular diseases. This article is novel and could yet help find sustainable means of managing PTSD among veterans.

Response*:* Thank you for your positive feedback. We hope the revised manuscript further enhances the novelty of this work.

**Reviewer #3**

**Comment:** Consider including more details about the machine learning techniques or predictive models used (or planned for future use) to analyze the collected data, as this is mentioned in the abstract but not elaborated upon in the methods or results sections.

Response*:* This study sought to examine the feasibility of using smartphone and wearable technology to predict PTSD symptom escalation and cannabis use among recently discharged veterans. This study does not present the use of machine learning, although further work may seek to do so. We have updated the Introduction and Discussion to highlight future opportunities.

**Comment:** The recruitment flowchart (Figure 1) is informative, but the reasons for ineligibility (e.g., "Multiple reasons") could be further clarified to enhance transparency.

Response*:* The “Multiple reasons” category in the flowchart represents when a participant has given a number of reasons for withdrawal, to ensure readability and a matching denominator, these have been aggregated. However, the categories are represented in the flowchart.

**Comment:** The retention rates are commendable, but the manuscript should address potential biases introduced by attrition, particularly in the later months of the study (e.g., 72% retention at 3 months).

Response*:* We now explicitly acknowledge the possibility of attrition bias. This is covered in the Discussion section (Discussion, page 17). We have also made additions following other Reviewer comments.

**Comment:** The variability in passive data completion rates (e.g., high for steps/distance but low for SPO2/respiration) is noteworthy. The discussion could explore potential reasons for these discrepancies (e.g., device-specific limitations, participant compliance) and their implications for future studies.

Response*:* We do not have enough information to accurate summarise our reasons as to why this may be the case. We hope to explore this in future work.

**Comment:** Clarify whether the "126 gigabytes of compressed data" refers to raw or processed data, and how this volume translates into analyzable datasets.

*Response:* We now clarify that the 126GB figure refers to raw compressed data across all participants, including wearable sensor streams and app-generated logs (Methods, page 15).

**Reviewer #4**

**Comment:**  this is a high quality pilot style project. The concept is interesting and may lead to a meaningful application of machine Learning in predicting the course and outcomes of THC use in PTSD and veterans.

Response*:* Thank you for your positive feedback. We hope the revised manuscript further enhances the novelty of this work. As the Reviewer comments are brief, we have attempted to distil the key points below.

**Comment:** Differences across wearable devices

Response*:* We now report on participants’ smartphone type, noting that the majority used Apple devices. While we do not report individual wearable device brands due to the heterogeneity of devices used, we note that 32 participants were provided with a Fitbit by the research team. These points are discussed in the Results (Results, page 14; Results, page 22).

**Comment:** MAVERICK data collection software

Response*:* We have expanded our discission of the MAVERICK software. This is included in the revised Methods (Methods, page 7). We are mindful of the length of the manuscript and have sought to keep the addition brief.

**Comment: Clarify our machine learning strategy**

Response*:* The manuscript now includes a clearer explanation of how the pilot data informs our ultimate aim, to develop predictive models for PTSD symptom changes related to cannabis use. This has been addressed as a result of Reviewer 1 comments.

We hope that our revisions satisfactorily address all comments. Thank you again for your valuable feedback and the opportunity to improve our manuscript.

Sincerely,

Dr Daniel Leightley

On behalf of all co-authors
